# Harms associated with taking nalmefene for substance use and impulse control disorders: A systematic review and meta-analysis of randomised controlled trials

**DOI:** 10.1371/journal.pone.0183821

**Published:** 2017-08-29

**Authors:** Karina Glies Vincents Johansen, Simon Tarp, Arne Astrup, Hans Lund, Anne K. Pagsberg, Robin Christensen

**Affiliations:** 1 Musculoskeletal Statistics Unit, The Parker Institute, Copenhagen University Hospital, Bispebjerg and Frederiksberg, Copenhagen, Denmark; 2 Department of Nutrition, Exercise and Sports, University of Copenhagen, Copenhagen, Denmark; 3 Institute of Sports Science and Clinical Biomechanics, University of Southern Denmark, Odense, Denmark; 4 Centre for Evidence-based Practice, Bergen University College, Bergen, Norway; 5 Child and Adolescent Mental Health Centre, Mental Health Services Capital Region of Denmark & Faculty of Health Science, University of Copenhagen, Copenhagen, Denmark; University of Toronto, CANADA

## Abstract

**Importance:**

Nalmefene is a newly approved drug for alcohol use disorder, but the risk of harms has not been evaluated from empirical trial evidence.

**Objective:**

To assess the harm of nalmefene administered to individuals diagnosed with substance use or impulse control disorders by performing a systematic review and meta-analysis of randomised controlled trials.

**Data sources:**

A search was performed in Cochrane Central Register of Controlled Trials (CENTRAL, 2014), MEDLINE via PubMed (1950), EMBASE via Ovid (1974), and Clinicaltrials.gov through December 2014.

**Study selection:**

This study included only randomised controlled trials with placebo or active controls that administered nalmefene to adult individuals for treating impulse control and/or substance use disorders. Both published and unpublished randomised controlled trials were eligible for inclusion.

**Data extraction and synthesis:**

Internal validity was assessed using the Cochrane risk-of-bias tool. Published information from the trials was supplemented by contact between reviewers and industry sponsor. Data were combined using two meta-approaches in fixed effects models; Peto Odds Ratios and risk differences were reported with 95% confidence intervals (95%CIs).

**Main outcomes and measures:**

Number of patients with serious adverse events, including specific psychiatric serious adverse events and withdrawals due to adverse events.

**Results:**

Of 20 potentially relevant studies, 15 randomised controlled trials met the inclusion criteria, and 8 of these provided data enabling the meta-analysis. Overall, serious adverse events did not occur more often in the nalmefene group than in the placebo group (Peto Odds Ratio = 0.97 [95% CI 0.64–1.44]; P = 0.86). Risk of psychiatric serious adverse events was slightly elevated, albeit not at a statistically significant level (Peto Odds Ratio = 1.32 [95% CI 0.62, 2.83]; P = 0.47). Withdrawals due to adverse events were significantly more likely to occur with nalmefene compared to placebo (Peto Odds Ratio = 3.22 [95% CI 2.46–4.22]; P<0.001)

**Conclusions and relevance:**

The three-fold increased risk of withdrawal from treatment on nalmefene due to adverse events is a matter of safety concern. The nature of these adverse events cannot be elucidated further without access to individual patients data.

## Introduction

Impulse control and substance use disorders are diagnostically separate mental illnesses that, however, share similar features of pathological behaviours [1]. Impulse control disorder (which include kleptomania, pathological gambling, pyromania, and intermittent explosive disorder) are “behavioural addictions” characterized by behaviours and urges that are harmful to oneself and impair social and occupational function as well as incur legal and financial difficulties [2,3]. Substance use disorder is an addiction characterized by a craving or a strong desire or urge to use a substance (e.g., drug, alcohol, or nicotine), which leads to clinically significant impairment or distress [4]. DSM-5 regards impulse control and substance use problems as disorders [4], and pharmacological intervention (e.g., with opioid antagonists) is one of the suggested opportunities for treatment [4].

Because adverse reactions from prescription drugs are so frequent, leading to 4.7% hospital admissions in the US, pharmacological treatment of impulse control and substance use disorders may lead one to infer adverse or serious adverse events (SAEs). An adverse event is considered serious by the investigator and sponsor of the trial if any of the following outcomes occur: death, a life-threatening adverse event, inpatient hospitalization or prolongation of existing hospitalization, a persistent or significant incapacity or substantial disruption of the ability to conduct normal life functions, or a congenital anomaly/birth defect [5]. In 1998, 2.7 million hospitalized Americans experienced SAEs from prescription drugs [6]. Furthermore, mortality from adverse drug reactions is 0.32%, constituting the fourth leading cause of death in US hospitals [7,8]. Nalmefene hydrochloride dehydrate (nalmefene) is an opioid antagonist and was approved by the European Medicines Agency (EMA) in 2012 to reduce alcohol consumption in patients with alcohol dependence. To date, no systematic review and meta-analysis has been performed regarding harm (SAEs psychiatric SAE or withdrawal due to adverse events (WD d/t AE) associated with use of nalmefene for substance use and impulse control disorders. Most of the systematic reviews investigate the effect of treating alcohol dependence with different drugs [9–16] without addressing the potential harm (e.g., SAEs and WD d/t AE). According to the EMA's nalmefene assessment report, oral nalmefene 18 mg/day is a well-tolerated and efficacious dose and overall, there were no SAEs causing major safety concern [17]. The report emphasizes that individuals with depressive or psychotic comorbidities were excluded from the different clinical trials investigating nalmefene; estimates of the potential psychiatric SAEs of the drug, therefore, are likely conservative [17–20].

In light of the likely increase in drug prescriptions and after approval of nalmefene by EMA (currently no marketing authorization request to the US Food and Drug Administration), we performed a systematic review and meta-analysis of RCTs comparing nalmefene with placebo or active controls to assess nalmefene's potential for causing harms.

## Methods

The definition of harm is “the totality of possible adverse consequences of an intervention or therapy; they are the direct opposite of benefits, against which they must be compared”[5]. Harms in the present study refer to SAEs, psychiatric SAEs or WD d/t AE.

An event refers to the overall number of SAE, psychiatric SAE or WD d/t AE, in the studies and is thus not necessarily an expression of any pathological causality between nalmefene and a specific effect.

The systematic review and the meta-analysis were performed according to the ‘*Preferred Reporting Items for Systematic reviews and Meta-Analyses*’ (PRISMA) guidelines for reporting systematic reviews and meta-analyses [21]. The protocol is registered and available on PROSPERO (CRD42014015279).

### Selection criteria and search strategy

This study included RCTs with placebo or active controls that administered nalmefene to individuals for treating impulse control and/or substance use disorders. We sought all placebo- and active-controls RCTs of nalmefene at any dose for any duration in humans. Both published and unpublished RCTs were eligible for inclusion in the systematic review. If the RCTs had data on harms, the RCTs were eligible for inclusion in the meta-analysis.

Online clinical registries and four bibliographic databases—Cochrane Central Register of Controlled Trials (CENTRAL, (2014); MEDLINE via PubMed (1950); EMBASE via Ovid (1974); and Clinicaltrials.gov—were searched through December 2014 for RCTs investigating the effect and safety of nalmefene compared to any control group. The search strategy is available from the protocol **S1 File**.

Online documents available from EMA regarding nalmefene were identified and scrutinized for data not available in scientific publications. All RCTs relating nalmefene to substance use or impulse control disorders were identified. Furthermore, the reference lists of all apparently relevant review articles and included studies were manually searched to identify other potentially relevant RCTs. One reviewer (KGVJ) screened all references by titles and abstracts according to the eligibility criteria. Full text papers were retrieved for further assessment if the abstract information suggested that the study compared nalmefene with placebo or an active comparator and included participants with impulse control or substance use disorders. Two reviewers (KGVJ, ST) assessed full-text papers for the systematic review.

### Data collection, data items, and risk-of-bias assessment

Data were extracted by one reviewer (KGVJ) and confirmed by a second reviewer (ST). Primary outcome measures were extracted as the overall number of nonspecific SAEs, defined by the International Conference on Harmonization of Technical Requirements for Registration of Pharmaceuticals for Human Use events [5], both in the nalmefene and in the control groups. Secondary outcomes are specific SAEs (e.g. all cause mortality) and specific psychiatric SAEs emphasizing depression, anxiety, suicidal attempts, suicidal ideation, and suicide. Furthermore, data were extracted on WD d/t AE. For exploratory qualitative purposes, all types of SAEs reported in the different trials were also extracted. We included all the aforementioned outcomes regardless of whether or not we believed that SAEs, psychiatric SAEs or WD d/t AE were related or not to drug treatment.

The Cochrane risk-of-bias tool was used to assess whether there was high, low, or unclear risk of bias in the following domains: sequence generation, allocation concealment, blinding, incomplete outcome data, and selective outcome reporting [22]. KGVJ and ST assessed the risk of bias independently for all included studies. Discrepancies were resolved by referring to the original publications and discussing with a third reviewer (RC). Study characteristics based on condition, trial duration, and number of participants (including doses and the percentage of males participating) were abstracted from the eligible trials.

### Statistical analyses

We described study characteristics according to sample size and characteristics of study participants, including mental illness condition (e.g. substance use disorder or impulse control disorder) and trial duration. Review Manager (5.3) was used to conduct all the meta-analyses. Because our outcomes of interest are rare, we followed recommendations of Bradburn and colleagues [23] and used Peto odds ratios (PORs) under fixed effects models to compare nalmefene and placebo/active comparator groups. We also undertook meta-analyses using Mantel-Haenszel risk differences (RD) under fixed effects models. We present results using both a relative measure as PORs and an absolute measure as RD. We reported results using 95% confidence intervals (95%CIs) and forest plots for both measures so that findings can be compared. Statistical heterogeneity was assessed with the I-squared statistic [24]; we assigned adjectives of low, moderate, and high to I^2^ values of 25%, 50%, and 75%, respectively [25]. A relevant study-level covariate was defined a priori as one that was able to decrease the between-study variance (tau-squared, and thus the I-squared statistic)[24]. Risk of bias across RCTs was assessed by using stratified analyses for each of the domains included in the risk-of-bias tool [22].

## Patient involvement

No patients were involved in setting the research question or the outcome measures, nor were they involved in the design and implementation of the study and there are no plans to involve the patients in the dissemination of the results.

## Results

**Fig 1:**
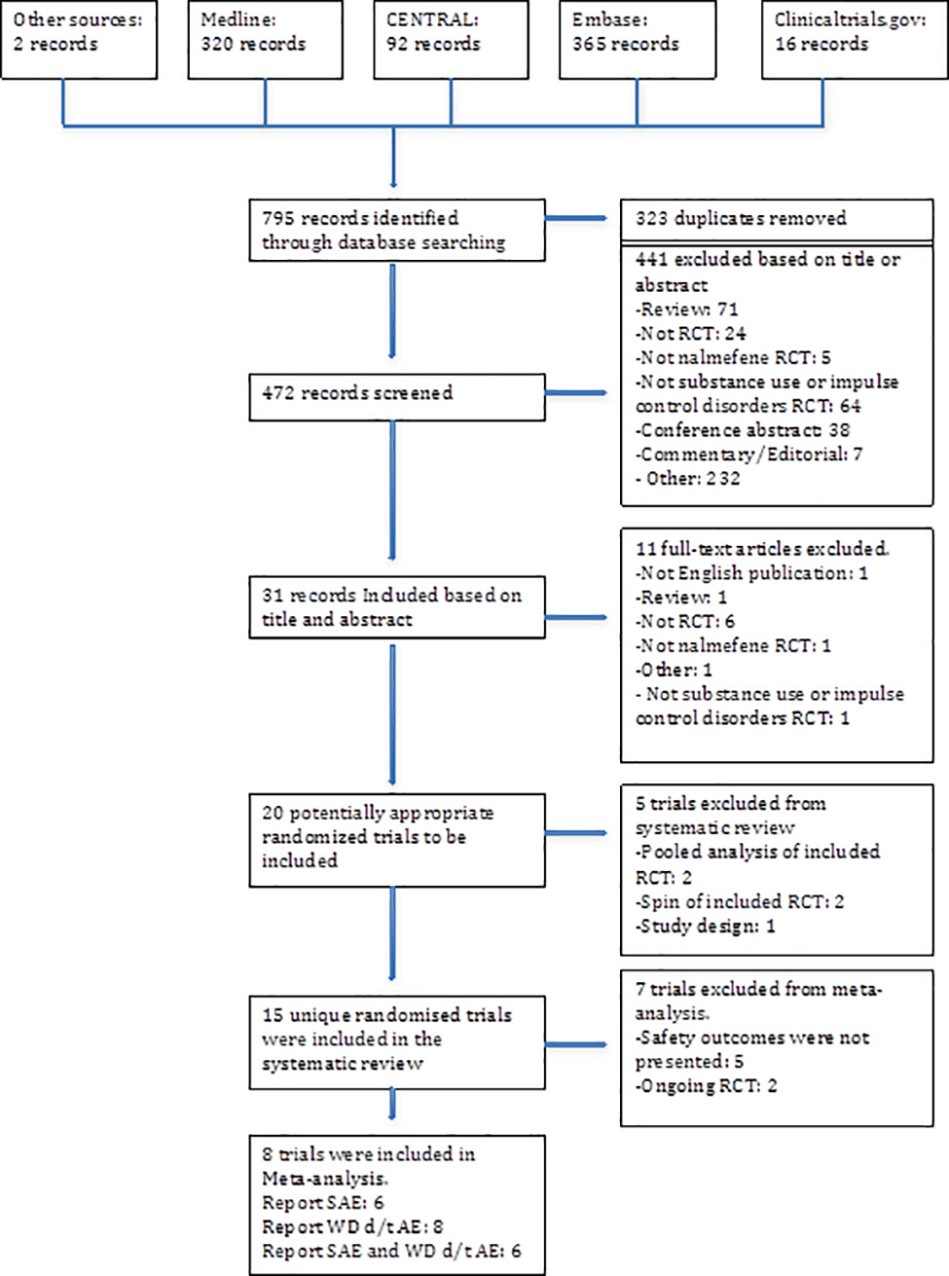
Flow chart showing selection of randomized controlled trials for inclusion in systematic review of nalmefene.

The bibliographic searches identified 795 references, and after removal of 323 duplicates across the different data sources, 472 titles and abstracts were screened (**Fig 1).** From these, 441 records were excluded based on abstract and title (93%). Of the remaining 31 records **S2 File**, a further 11 full-text articles were excluded based on the eligibility criteria. Thus, 20 records were identified as potentially eligible RCTs retrieved for further scrutiny **S2 File**. Two of the 20 records [26,26,27] were spinoff papers of two already included RCTs [19,28]. One record that was simply excluded from the study since the record wasn’t an RCT [29,29], and two other RCTs were pooled analyses of included RCTs [30,31].

Ultimately, eight studies were used as the basis of the statistics on harms from nalmefene [19,20,32–37]. 15 fulfilled eligibility requirements [18–20,28,32–38,38–41] but were further whittled down to 8 because of missing data on harms.

**Table 1** illustrates the study characteristics and the risk-of-bias assessment of the 15 RCTs included in the systematic review. From these 15 RCTs, two were ongoing studies [39,41].

**Table 1:** Study characteristics including the risk of bias assessment of randomised controlled trials of nalmefene included in the systematic review.

Author, Year, (Ref)	Outcomes Included	Condition	Trial duration	No. of patients	Male	Mean age	Risk of bias	Opioid antagonist	Daily dose	No. of patients
Included in the meta-analysis*	in meta-analysis		(Weeks)	randomised	(%)	year	Δ		(mg)	
Anton et al 2004	WD d/t AE	Alcohol Use Disorder	12	270	210	45.0	U/U/U/U/I	Nalmefene	40	68
CPH-101-0299					(78)			Nalmefene	20	66
[35](*)								Nalmefene	5	68
								Placebo	0	68
Grant et al 2006	WD d/t AE	Gambling Disorder	16	207	116	45.9	A/U/U/U/I	Nalmefene	100	52
[20](*)					(56)			Nalmefene	50	52
								Nalmefene	25	52
								Placebo	0	51
Gual et al 2013	SAE	Alcohol Use Disorder	24	718	503	44.8	A/A/A/I/A	Nalmefene	18	358
NCT 00812461	Anxiety				(70)			Placebo	0	360
(ESENSE 2)	Depression									
[37](*)	WD d/t AE									
Karhuvaara et al 2007	SAE	Alcohol Use Disorder	28	403	326	49.2	A/A/A/A/U	Nalmefene	10–40	242
CPH-101-0801	WD d/t AE				(81)			Placebo	0	161
[19](*)										
Mann et al 2013	SAE	Alcohol Use Disorder	24	604	405	51.6	A/A/A/I/A	Nalmefene	18	306
NCT00811720	Anxiety				(67)			Placebo	0	298
(ESENSE 1	Depression									
[36](*)	WD d/t AE									
Mason et al.1994	SAE	Alcohol Use Disorder	12	21	15	42.0	U/A/U/U/A	Nalmefene	40	7
[34](*)	WD d/t AE				(71)			Nalmefene	10	7
								Placebo	0	7
Mason et al 1999	SAE	Alcohol Use Disorder	12	105	70	41.8	U/A/U/A/A	Nalmefene	20–80	70
[33](*)	WD d/t AE				(67)			Placebo	0	35
Van den brink et al 2014	SAE	Alcohol Use Disorder	52	675	506	44.3	A/A/A/I/A	Nalmefene	18	499
(SENSE)	Anxiety				(75)			Placebo	0	163
NCT00811941	Depression									
[32](*)	WD d/t AE									
Grant et al 2010	NA	Gambling Disorder	10	233	135	46.5	A/U/U/U/I	Nalmefene	40	82
[18]					(58)			Nalmefene	20	77
								Placebo	0	74
Drobes et al.2000	NA	Alcohol Use Disorder	1	25	20	35.1	U/U/U/U/I	Naltrexone	NA	NA
[40]					(80)			Nalmefene	NA	NA
								Placebo	0	NA
Drobes et al.2003	NA	Group a)	1	125	NA	30.3	A/U/U/U/I	Naltrexone	25–50	39
[28]		Alcohol Use Disorder						Nalmefene	20–40	36
								Placebo	0	50
		Group b)	1	90	NA	32.0	A/U/U/U/I	Nalmefene	25–50	29
		social drinkers						Nalmefene	20–40	31
								Placebo	0	30
Somaxon pharmaceuticals	NA/on	Smoking Use Disorder	5	NA	NA	NA	NA	Nalmefene	80	NA
[39]								Nalmefene	40	NA
								Placebo	0	NA
Lundbeck A/S 2014	NA/on	Alcohol Use Disorder	1+1day	100	NA	NA	NA	Nalmefene	18	NA
[41]								Placebo	0	NA
					
Biotie	NA	Alcohol Use Disorder	28	167	NA	NA	NA	Nalmefene	40	82/3
CPH-101-0701								Nalmefene	20	NA
[38]								Nalmefene	10	NA
								Placebo	NA	85
Biotie	NA	Alcohol Use Disorder	16	150	NA	NA	NA	Nalmefene	40	50
CPH-101-0399								Nalmefene	10	50
[38]								Placebo	0	50

**Δ**Risk of bias: sequence generation/concealment of allocation/blinding of participants and investigators/incomplete outcome data/selective outcome data. A = Adequate (low risk of bias); U = Unclear (unclear risk of bias); I = Inadequate (high risk of bias). NA = data not available. DSM-5 = Diagnostic and Statistical Manual of Mental Disorders. SAE = serious adverse event. WD d/t AE = withdrawal due to adverse event.

* Included in the meta-analysis.

The remaining 13 completed RCTs, undertaken between 1994 and 2014, were multicenter studies, with a total of 3,793 participants (based on the intention-to-treat populations) assessed. The mean age for participants in the included studies ranged from 30.3 to 51.6 years. In the included studies, most of the participants were male; the majority ranged from 58 to 81%. Trial durations varied from one day to 52 weeks, and the participants received either 5–100 mg/day nalmefene or 25–50 mg/day of naltrexone, or placebo. Of the 15 completed RCTs, five did not report on SAEs or WD d/t AE [17,18,28,38,38,40]. Eight RCTs were included in the meta-analysis [19,20,32–37]. All eight RCTs reported WD d/t AE. Six RCTs, reported both on SAEs and WD d/t AE [19,32–34,36,37]. Two of these eight RCTs did not report on SAEs, and data could not be identified through other sources; hence we deemed these two RCTs [20,35] a high risk of selective-outcome-reporting bias. Three of the eight RCTs used as-treated data instead of intention-to-treat data and therefore were considered high risk of incomplete-data bias [32,36,37].

The objectives of the available studies in the meta-analysis were to examine the safety and efficacy of nalmefene for reducing alcohol consumption in alcohol-dependent patients [33–37], to evaluate the long-term efficacy and safety of nalmefene in reducing alcohol consumption [32], to determine the effect and safety of nalmefene in reducing heavy drinking [19], and to examine the efficacy and tolerability/safety of nalmefene in reducing of pathological gambling [20].

### Harms observed

**Fig 2:**
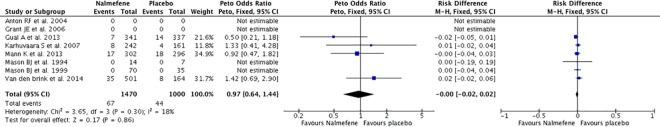
Serious adverse events of nalmefene compared to placebo in patients with substance use or/and impulse control disorder.

Six of the ultimately eight RCTs that were used as the basis of the statistics on harms from nalmefene reported SAEs (**Fig 2).** Forest plot of odds (Peto odds ratio) and risk (Mantel-Haenszel risk difference) of serious adverse event associated with nalmefene use compared to placebo. Every square represents the individual study’s serious adverse events estimates with 95% CI indicated by horizontal line. We found no evidence of increased odds of SAEs in the nalmefene group compared with the placebo group. The Peto Odds (POR) for nalmefene versus placebo was POR 0.97 (95% CI 0.64 to 1.44; P = 0.86; I^2^ = 18%), and the Risk difference (RD) was -0.00 (-0.02 to 0.01; P = 0.84; I^2^ = 0%). There was no reason to suspect a rejection of the hypothesis of homogeneity based on the P value in the POR method Q = 3.65; P = 0.30.

Three out of the six RCTs that reported SAEs categorized SAEs into different sub-disorders such as psychiatric disorders or death. To analyse secondary harm outcomes, we used the overall number of psychiatric SAEs **S1 Fig**; the POR for nalmefene versus placebo was POR 1.32 (95% CI 0.62 to 2.83; P = 0.47, I^2^ = 40%). The RD was 0.00 (-0.01 to 0.01; P = 0.40, I^2^ = 72%). In other words, we found no evidence of increased odds of overall psychiatric SAEs in the nalmefene group compared with the placebo group. Based on the Cochrane Q statistic test, homogeneity among the studies was present according to the p-value in the POR method Q = 3.33, P = 0.19. However, the analysis showed a moderate degree of inconsistency among the study findings according to their I^2^ values.

After we have analysed the overall number of psychiatric SAEs we subcategorized psychiatric SAEs into depression, anxiety, and mortality (including completed suicide and sudden death). Of the six studies mentioned above, three reported depression; the POR was 5.48 (95% CI 0.28 to 106.79; P = 0.26, I^2^ = 0%). The RD was 0.00 (-0.01 to 0.01; P = 0.60, I^2^ = 0%). For anxiety; the POR was 3.77 (95% CI 0.04 to 355.81; P = 0.57, I^2^ = Not applicable. Hence, only one study reported events on anxiety). The RD was 0.00 (-0.00 to 0.00; P = 0.87, I^2^ = 0%). Mortality, including suicide and sudden death, was reported in three RCTs (total 1/1144) in the nalmefene group and 2/797 in the placebo group). The POR for mortality was 0.50 (95% CI 0.05 to 4.85; p = 0.55, I^2^; = 63%) and the RD was -0.00 (-0.01 to 0.01; P = 0.93, I^2^ = 0%). Based on the Cochrane Q statistic test, homogeneity among the studies was present Q = 2.68; P = 0.10. We found no evidence of an increased risk of depression, anxiety, or death in the nalmefene group compared with the placebo group. A list of all different reported SAEs in the included studies is presented in **S1 Table.**

**Fig 3:**
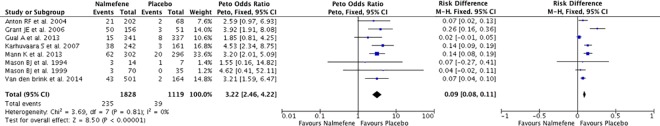
Withdrawals due to adverse events of nalmefene compared to placebo in patients with substance use or/and impulse control.

Eight RCTs in the meta-analysis reported WD d/t AE (**Fig 3).** Forest plot of odds (Peto odds ratio) and risk (Mantel-Haenszel risk difference) of withdrawals due to adverse events associated with nalmefene use compared to placebo. Every square represents the individual study’s withdrawals due to adverse events estimates with 95% CI indicated by horizontal line; the POR was 3.22 (95% CI 2.46 to 4.22; P<0.00 I^2^; = 0%). The RD was 0.09 (0.08 to 0.11; P = 0.00, I^2^ = 86%). Based on the Cochrane Q statistic test, homogeneity among the studies was present according to the p-value in the POR method Q = 3.69; P = 0.81.

### Additional analyses

The risk of bias across RCTs according to the major outcome (SAE) was assessed by using subgroup analyses for each of the five domains: sequence generation, allocation concealment, blinding, incomplete outcome data, and selective outcome reporting stated in the Cochrane risk-of-bias tool, including if the RCTs were using the as-treated or intention-to-treat principle **S2 Table**. The subgroups' differences for sequence generation, allocation concealment, and blinding were not applicable because all the included studies provide data only in the adequate categorizations in the above mention domains. For Intention to treat (P = 0.57), incomplete outcome data (P = 0.57), and selective outcome reporting (P = 0.57), the differences among the groups were not significant.

Even if there are similarities as indicated in the diagnostics, the abuse of alcohol can very well have changed or modulated biology so that response to a drug and also potential WD t/t AE. Since the risk of WD d/t AE was more than 3 times likely for the patient in the nalmefene group than for the placebo group, a subgroup analysis stratified in two groups, substance use disorder and impulse control disorder was made **S2 Fig.** The subgroup differences for the two groups were P = 0.57; I^2^ = 0%, the differences between the two groups were not significant.

## Discussion

This meta-analysis of eight RCTs involving 1,828 patients with substance use or impulse control disorder who were prescribed nalmefene and 1,119 patients who were prescribed placebo found no increased risk of SAEs compared to placebo. However, patients taking nalmefene were 3.22 times more likely to discontinue therapy due to side effects compared to placebo. In the WD d/t AE stratified analysis, no statistical differences among the two groups were observed.

Treatment with nalmefene did not increase risk of death when compared to placebo. The overall psychiatric SAEs apparently occurred more frequently, but not statistically significantly in the nalmefene group compared to placebo. In the risk-of-bias stratified analyses, no statistical differences among the different groups in the risk-of-bias tool were observed.

### Comparison with other studies

Currently there is no systematic review and meta-analysis report on SAEs associated with use of nalmefene for substance use or impulse control disorders. Two meta-analyses by Jones et al. and Rösner et al. reported on the effect of different pharmaceutical treatments for alcohol dependence and included an analysis of WD d/t AE [10,15]. Jonas et al. included 135 studies, seven of which compared nalmefene with placebo [10,11]. Compared with placebo, participants treated with nalmefene or naltrexone had a higher risk of WD d/t AE. In one study two participants in placebo group committed suicide. In another trial one patient allocated to the placebo group died of hepatocellular carcinoma and one patient randomised to nalmefene died suddenly for unknown causes. In studies comparing nalmefene with placebo, four cases of suicide attempts or suicidal ideation in nalmefene group and 9 in placebo group were reported. Rösner et al. included 50 RCTs, including three studies comparing nalmefene to placebo (13). Compared to the placebo group, patients in the nalmefene group had a 43% higher risk of WD d/t AE. The dropout rates were not statistically significant [15]. SAEs were not reported as an outcome or calculated in that meta-analysis. Our meta-analysis has shown a similar trend according to withdrawals due to adverse events in the nalmefene group compared to placebo group.

### Strengths and limitations

To the best of our knowledge, ours is the most comprehensive systematic review and meta-analysis of harm associated with using nalmefene to treat impulse control and substance use disorders.

Our systematic review was rigorously performed and reported according to the PRISMA statement [21]. A highly sensitive search strategy was used in order to identify as many relevant studies as possible and to reduce potential publication bias. An extensive range of resources was searched, including electronic databases, guidelines, and references listed in other systematic reviews. Both published and unpublished RCTs were eligible for inclusion in the systematic review and if the RCTs had data on harms, the RCTs were eligible for inclusion in the meta-analysis. We present results using both a relative measure as Peto Odds Ratio and an absolute measure as Risk Difference with a fixed effect model. We used the Cochrane risk-of-bias tool to assess the internal validity in all the included studies.

Our results were limited by a lack of access to all available sources reporting safety data, including unpublished RCTs and RCTs in progress, as well an absence in several trials of consistent reporting of psychiatric SAEs. Additionally, the secondary endpoints reported in the studies were depression, anxiety, suicidal attempts, suicidal ideation, and suicide and mortality; some of these phenomena had very different severity and clinical implications. In all the studies, the enrolled patient populations were highly selected, as patients with a history of comorbid psychiatric disorder were excluded from the studies. For these reasons, our estimates of psychiatric SAEs are probably conservative, and our results cannot be extended to populations of patients with substance use or impulse control disorders and other psychiatric comorbidity.

Many of the RCTs included in the meta-analysis were relatively small and short-term, resulting in few events; our study, therefore, was not able to effectively discuss the risk for further SAEs in larger studies. The limited number of events resulted in PORs that could be affected by small changes in the classification of events and may have caused problems concerning heterogeneity in the meta-analysis. The confidence intervals for the PORs for SAEs, WD d/t AE, and deaths were wide, resulting in considerable uncertainty about the magnitude of the observed odds.

We did not have full access to all original source data, and we based the meta-analysis on available data from publications. However, we found two RCTs in the Selincro assessment report [17] and in the Biotie Therapies Corp. update [38]. These RCTs had not yet been published; hence, we weren’t able to include them in the meta-analysis together with the risk-of-bias analysis. Two RCTs had not been completed—one for using nalmefene for smoking cessation and one for using nalmefene to treat alcohol dependence [39,41]. Ongoing RCTs may provide useful data, which could have increased the probability for significance within our primary and/or secondary results had it been possible to include them [39,41].

According to our objective, the two domains—*selective outcome reporting* and *incomplete outcome data*—were both considered as important domains in the risk-of-bias tool. Based on these tools, five RCTs had high risk of bias from selective outcome reporting and an unclear risk of bias from incomplete outcome data. Another three RCTs had a high risk of bias from incomplete outcome data, resulting in as-treated data's being used. The proportion of information from RCTs at high risk of bias in the key domains was sufficient to affect the interpretation and validity of the result. Some of the results were not applicable because some outcomes were not estimable in some of the groups, which decreased the reliability of our study's results.

The manufacturers' and authors' public disclosure of safety results for nalmefene RCTs were not sufficient to enable a robust assessment of specifically the risk of SAEs from nalmefene. The manufacturers and authors have available all source data for completed RCTs and should make these data available due to ethical considerations. If more manufacturers and authors chose to strictly follow good clinical practice (GCP), harmonization of technical requirements for registration of pharmaceuticals for Human Use (ICH) and the CONSORT Statement for better reporting of harm in randomised trials [5], then market transparency will increase, resulting in greater chances for improved validity.

### Conclusions and clinical implications

Based on our systematic review of published RCTs, there is no evidence of an increased risk of SAEs including psychiatric SAEs and death from any cause associated with treatment with nalmefene for substance use and impulse control disorders. Nalmefene users, however, have an increased risk of WD d/t AE, which are consistent with findings from other systematic reviews. However, this conclusion is based upon a highly selected group of patients; a more representative selection procedure may not rule out the possibility of more adverse events. This risk seems to be supported by an “increased risk of WD d/t AE," which likely reflects the ultimate decision of the participant and physician to quit the intervention due to side effects.

The findings of our meta-analysis suggest a risk of WD d/t AE associated with use of nalmefene compared to placebo, but the risk of SAEs remains unclear until more precise estimates of SAEs associated with nalmefene treatment in patients suffering from substance use or impulse control disorder has become available. Further studies are needed to enable a more robust assessment of the harm of nalmefene. Large RCTs with more participants in both groups and a balance in the number of participants in the groups, comparing placebo with nalmefene, reporting on both SAEs and WD d/t AE, with use of Intention-to-treat data are strongly recommended to strengthen the evidence base for treatment with nalmefene.

## Supporting information

S1 File Trial protocol—Harms associated with use of nalmefene for substance use and impulse control disorder: Protocol for a systematic review and meta-analysis of randomised trials. (PDF)

S2 File Reference list of all 31 full text papers included based on title and abstract. (DOCX)

S3 File PRISMA-Checklist. (DOC)

S1 Table Exploratory table of all serious adverse events reported in all the selective studies. (DOCX)

S2 Table Results of risk-of-bias stratified meta-analyses for serious adverse event(s) (Peto Odds Ratio). * NE = not estimable. NA = Not applicable.
(DOCX)

S3 Table Available data set *NA = Not applicable. (DOCX)

S1 Fig Psychiatric serious adverse events on nalmefene compared to placebo in patients with substance use or/and impulse control disorder. (TIF)

S2 Fig Subgroup analysis of withdrawal due to adverse events. (PNG)
