# Supplementary material for: Harms associated with taking nalmefene for substance use and impulse control disorders: A systematic review and meta-analysis of randomised controlled trials
Source: PLoS One. 2017 Aug 29;12(8):e0183821. doi: 10.1371/journal.pone.0183821 (PMC5574613; doi:10.1371/journal.pone.0183821)
Supplement: S1 File — (PDF) [file pone.0183821.s001.pdf]

# Harms associated with use of nalmefene for substance use and impulse control disorders:

***Protocol for a systematic review and meta-analysis of randomised trials***

K. G. V. Johansen[1,2], S. Tarp [1], A. Astrup[3], H. Lund[4], A. K. Pagsberg[5], R. Christensen[1]

1: Musculoskeletal Statistics Unit, The Parker Institute, Department of Rheumatology, Copenhagen University Hospital, Bispebjerg and Frederiksberg, Copenhagen, Denmark.

2: Faculty of health science, University of Southern Denmark, Campus Esbjerg, Denmark

3: Department of Nutrition, Exercise and Sports, University of Copenhagen, Copenhagen, Denmark

4: Institute of Sports Science and Clinical Biomechanics, University of Southern Denmark, Odense, Denmark.

5: Mental Health Services-Capital Region of Denmark and Faculty of Health Science, Child and Adolescent Mental Health Center, University of Copenhagen Denmark, Denmark.

**Correspondence to:**

Robin Christensen, MSc, PhD. (Senior biostatistician)
Professor of Clinical Epidemiology.

Head of Musculoskeletal Statistics Unit (MSU)
The Parker Institute, Department of Rheumatology,
Copenhagen University Hospital, Bispebjerg and Frederiksberg
Nordre Fasanvej 57
DK-2000 Copenhagen F
Denmark
E-mail: Robin.Christensen@frh.regionh.dk;
Tel: (+45) 3816 4165;
Fax: (+45) 3816 4159

**Keywords:** opioid antagonist, Nalmefene, serious adverse events, meta-analysis

**Word count (excluding title page, abstract and references):** 18686

**Tables/Figures:** 1/0

# ABSTRACT

## Introduction

To manage impulse control and substance use disorder pharmacological procedures such as nalmefene is now one of the suggested opportunities. By using pharmacological procedures as treatments adverse or serious adverse event may occur. Adverse drug reactions from prescription drugs lead to 4.7 % hospital admissions in the US. The mortality of adverse drug reactions is 0.32 %, which leads to the 4$^{th}$ leading cause of deaths in hospitals in the US.

## Methods and analyses

We will follow a standard protocol for all review steps. Our manuscript will conform to the '*Preferred Reporting Items for Systematic reviews and Meta-Analyses*' (PRISMA) guidelines for reporting systematic reviews and meta-analyses.

We will search the following bibliographic databases; the Cochrane Central Register of Controlled Trials (CENTRAL, latest issue), MEDLINE via Pubmed (1950), EMBASE via Ovid, and Clinicaltrials.gov.

## Ethics and dissemination

As no primary data collection will be undertaken, no additional formal ethical assessment and informed consent are required. Our goal is to help decision-makers and nalmefene drug prescribing physicians make evidence-based decisions, which will enable a comprehensive interpretation of the data for harm.

## Protocol registration

CRD42014015279.

# INTRODUCTION

## Description of the conditions

Impulse control disorder is a psychiatric condition, which is characterised by behaviours and urges that are harmful to one self and affect individual's impairment in social and occupational functions as well as incur legal and financial difficulties (Grant and Potenza 2004;Schreiber et al. 2011). Diagnostic and statistical manual of mental disorder (DSM-5) classifies kleptomania, pathological gambling, pyromania and intermittent explosive disorder as impulse control disorders (American Psychiatric Association 2013;Schreiber, Odlaug, & Grant 2011). In ICD-10, Habit and Impulse Disorders, F63, includes certain disorders of behaviour that are characterized by repeated acts that have no clear rational motivation, cannot be controlled, and generally harm the patient's own interests and those of other people. The patient reports that the behaviour is associated with impulses to action. The cause of these disorders is not understood and they are grouped together because of broad descriptive similarities, not because they are known to share any other important features. F63 includes pathological gambling, pyromania, kleptomania, trichotillomania and other/unspecified habit and impulse disorders, but excludes habitual excessive use of alcohol or psychoactive substances (F10-F19) as well as impulse and habit disorders involving sexual behaviour (F65)(World Health Organization 2014). Substance use disorders is another psychiatric condition, which are characterised by a craving or a strong desire or urge to use a substance, which lead to clinically significant impairment or distress. Substance refers to drugs, alcohol and nicotine (American Psychiatric Association 2013).

To manage psychiatric conditions pharmacological procedures are now one of the suggested opportunities for treatments. Pharmacological treatments have been used to treat abstinence from different substance, like methadone and disulfiram. Later pharmacological treatments have focused on the addiction in the central nervous system. These pharmacological treatments include different drugs such as antidepressants, opioid agonists and opioid antagonists.

By using pharmacological procedures as treatments adverse or serious adverse event may occur. Adverse drug reactions from prescription drugs lead to 4.7 %

hospital admissions in the US. Furthermore in 1998, 2.7 million hospitalized Americans experienced a serious adverse drug reaction due to prescription drugs (Lazarou et al. 1998). The mortality of adverse drug reactions is 0.32 %, which lead to the 4th leading cause of deaths in hospitals in the US (Pirmohamed et al. 2004;van der Hooft et al. 2008).

A balance between impairment/distress of the body or social and financial difficulties according to substance use or impulse control disorders and adverse and serious adverse has to be considered in relation to use pharmacological procedures as treatments.

## Description of the intervention

Opioid antagonists for pharmacological treatment such as naloxone, naltrexone, and the recently competitive antagonist, nalmefene are tested and used to treat substance use and Impulse control in adults (Sadock and Sadock 2008;Schreiber, Odlaug, & Grant 2011;Soyka and Rosner 2010).

Nalmefene hydrochloride dehydrate was approved by the European Medicines Agency in 2012 to reduce alcohol consumption in patients with alcohol dependence. According to the EMA nalmefene assessment report, nalmefene 18 mg is well tolerated and efficacious (European Medicines Agency 2012). The investigators state that the safety profile appears consistent in and across all conducted clinical studies according to the updated version; summary of product characteristics (European Medicines Agency 2014). Nalmefene as medical treatment has been tested for pathological gambling, binge eating and eating disorders, reduction in total food intake and food preference (Cottone et al. 2008;de and Mitchell 1992;Kelly et al. 2014;Schreiber, Odlaug, & Grant 2011). For substance use disorders, nalmefene was tested for opiate use disorders (Fudala et al. 1991;Gual et al. 2013).

Although the safety profile appears consistent without any significant harm signals, serious adverse or adverse events still occurs in the assessment report. Three out of four (75%) participants have had adverse events following the nalmefene administration, compared to 62 % in the placebo group. Additionally,

study discontinuation according to intolerable adverse events in the nalmefene group was 10. 5 %. During the phase III trial programme two subjects committed suicide, however both of them being allocated to the placebo group. Two subjects committed self-harm or had suicidal behaviour, both of them being allocated to the nalmefene group (European Medicines Agency 2012). A former double blind, placebo-controlled pilot study and a human laboratory-based study questioning how plausible it is that opioid antagonists will result in central nervous system related adverse or serious adverse events emphasising depression and dysphoria. The investigators state that it is plausible that nalmefene compared to naltrexone is associated with greater reporting of side effects. Especially central nervous system related adverse or serious adverse events, such as dysphoria and cognitive disturbances (Drobes et al. 2003;Mason et al. 1994). The investigators in both studies concluded that further studies have to be made to confirm and state the findings (Drobes, Anton, Thomas, & Voronin 2003;Mason, Ritvo, Morgan, Salvato, Goldberg, Welch, & Mantero-Atienza 1994).

Subjects with depressive or psychotic co-morbidity were excluded from different clinical trials investigating nalmefene, hence the estimate of the potential psychiatric adverse or serious adverse events of the drug are conservative (European Medicines Agency 2012;Grant et al. 2006;Grant et al. 2010;Karhuvaara et al. 2007).

## Why it is important to do this review

In the light of the likely increase in drug use for impulsive control disorders and substance use disorders in the future and the anticipated adverse drug reactions, a critical systematic review and a meta-analysis would be relevant to investigate potential harms associated with nalmefene for impulsive control disorders and substance use disorders. Currently there is no systematic review and meta-analysis documenting the harm associated with use of nalmefene for substance use disorder or impulse control disorder. Most of the systematic review and the meta-analysis investigate the effect and benefit according to treat alcohol dependence with different drugs (table 1).

## Objectives

Our objective is to perform a systematic review and meta-analysis of randomised trials comparing nalmefene with placebo or active comparators to assess the effect of this agent on serious adverse events. The source material for the analysis consists of publicly available data from published trials as well as that submitted to health authorities including clinicaltrials.gov. The emphasis a priori is on psychiatric serious adverse events such as depression, anxiety and suicidal ideation/attempts(Christensen et al. 2007).

# METHODS

## Protocol and registration

We will follow a standard protocol for all review steps. Our protocol will be registered on PROSPERO (CRD42014015279); our manuscript will conform to the '*Preferred Reporting Items for Systematic reviews and Meta-Analyses*' (PRISMA) guidelines for reporting systematic reviews and meta-analyses (Liberati et al. 2009).

## Eligibility criteria

We will include all randomised controlled trials studying the effect of nalmefene on impulse control and substance use disorder. Trials comparing nalmefene to placebo or active comparator will be included. Publication date or publication status restrictions will not be imposed. Participants of any age and sex with a diagnosis of substance use or impulse control disorders will be included.

Any clinical trial phase (with a control group), dose levels, oral or intravenous of nalmefene, and length of follow-up or duration time, will be scrutinised for eligibility criteria.

**Types of outcome measures**

Primary outcome

The overall number of serious adverse events both in the nalmefene and in the control groups, according to studies investigating nalmefene treatment.

Serious adverse event is defined by International Conference on Harmonisation of Technical Requirements for Registration of Pharmaceuticals for Human Use (Ioannidis et al. 2004).

Secondary outcome

Serious adverse events, which emphasises:

*Depression*: Major depressive disorders both recurrent and single episode, emphasis will be on severe without psychotic features and/or severe with psychotic feature

*Anxiety:* Anxiety disorders include panic disorders, generalized anxiety, phobias and obsessive-compulsive disorders

*Suicidal attempts:* standardised Medical Dictionary for Regulatory Activities queries (SMQs) definition of 'suicide/self-injury'.

*Suicidal ideation: thinking about, considering or planning for suicide*

*Mortality:* all-cause mortality

Other psychiatric serious adverse events.

Withdrawal due to adverse events.

Exploratory secondary outcomes

An overview table will be conducted to collect and visualize all serious adverse events according to the trial reports.

## Information sources and search

We will search the following bibliographic databases; the Cochrane Central Register of Controlled Trials (CENTRAL, latest issue), MEDLINE via Pubmed (1950), EMBASE via Ovid (1974), and Clinicaltrials.gov. Online availably documents from EMA

regarding nalmefene will be identified and scrutinized for data not available in scientific publications. Relevant pharmaceutical company homepages will also be scrutinized for relevant information (e.g. clinical trial reports).

*The following search strategy will be applied in PubMed:*

("nalmefene" [Supplementary Concept]) OR 55096-26-9 OR ORF-11676 OR Nalmetrene OR JF-1 OR Cessal OR Soberal OR Alcofene OR Revex OR Arthene OR Cervene OR Incystene OR Selincro OR NIH-10365 (word variations have been searched).

*The following search strategy will be applied in EMBASE:*

(nalmefene or 55096-26-9 or ORF-11676 or Nalmetrene or JF-1 or Cessal or Soberal or Alcofene or Revex or Arthene or Cervene or Incystene or Selincro or NIH-10365).ti,ab.

*The following search strategy will be applied in Cochrane Central Register of Controlled Trials:*

#1: nalmefene OR 55096-26-9 OR ORF-11676 OR Nalmetrene OR JF-1 OR Cessal OR Soberal OR Alcofene OR Revex OR Arthene OR Cervene OR Incystene OR Selincro OR NIH-10365 (word variations have been searched).

*The following search strategy will be applied in Clinicaltrials.gov*

Study Type: Interventional studies

Interventions: nalmefene OR 55096-26-9 OR ORF-11676 OR Nalmetrene OR JF-1 OR Cessal OR Soberal OR Alcofene OR Revex OR Arthene OR Cervene OR Incystene OR Selincro OR NIH-10365

**Study selection**

One reviewer will start by screening titles and abstracts according to the inclusion criteria (KGVJ). Potentially relevant full text papers for the systematic review will be assessed by two systematic reviewers (KGVJ and ST) according to the eligibility

criteria if it seems relevant for the review question. The relevant papers, which obtain the eligibility criteria, will be used for the systematic review. To summarise the process of the study selection at PRISMA flow diagram will be generated (Liberati, Altman, Tetzlaff, Mulrow, Gotzsche, Ioannidis, Clarke, Devereaux, Kleijnen, & Moher 2009).

## Data collection process and data items

Data will be extracted by one author (KGVJ) and spot checked by a second reviewer (ST) on general characteristics of the RCT. Date of publication, type of journal, sample size, funding source (public, private, or unreported), inclusion and exclusion criteria and study design (parallel, cross, cluster or factorial) will be extracted.

Furthermore we will note whether the RCT was a single-centre or a multicentre trial. We will note the comparator and the dosage in the trials, and whether the individual RCT would be considered open-label, or masked. Corresponding authors from different trial reports and/or nalmefene drug manufacturers will be contacted for potential missing data.

## Risk of bias in individual studies

Two authors will assess the risk of bias, within each RCT using the domains of the risk-of-bias tool, as recommended by the Cochrane Collaboration (Higgins et al. 2011). Each included study based on the following domains with rating of low, high or unclear risk of bias will be investigated.

<u>Sequence generation</u>

The allocation sequence will be described in detail and judged to assess, whether the allocation sequence was adequately generated.

Allocation concealment

Allocation concealment will be described to assess whether the allocation was concealed.

Blinding

Any methods used to blind (*i*) participants, (*ii*) personnel and (*iii*) outcome assessors in the studies, will be described and judged, according to if knowledge of the allocated intervention was adequately prevented?

Incomplete outcome data

Main outcome data will be described and judged according to completeness and whether incomplete outcome data was adequately addressed (i.e., Intention to treat principle), including whether a flow diagram is available.

Selective outcome reporting

Outcome reporting will be evaluated to assess the possibility of selective outcome reporting, and whether the outcome reporting is free of suggestion of selective reporting.

## Summary measures and synthesis of results

We anticipate that many trials have few serious adverse events, so the odds ratios (OR) and 95% confidence intervals will be calculated with the use of the Peto method (Nissen and Wolski 2007). By using this approach, trials in which patients had no serious adverse events in either group will be excluded from analyses. We will also use exact methods for calculation of the 95% confidence intervals according to odds ratio, since asymptotic results can be unreliable when distribution of the dichotomous data is sparse (Christensen, Kristensen, Bartels, Bliddal, & Astrup 2007). To calculate the exact probabilities of the possible (2x2) tables we use Fisher´s exact test, which enable us to estimate the Wald test associated variance, corresponding to the ratio of its estimate ($log_e$-OR) to its standard error

(Christensen, Kristensen, Bartels, Bliddal, & Astrup 2007). Statistical heterogeneity across the various RCTs, will be tested by using the Cochran Q-test statistic (Cochran 1954). The result will be evaluated as the $I^2$ value, and interpreted as the percentage of the total variation across studies (Higgins, Altman, Gotzsche, Juni, Moher, Oxman, Savovic, Schulz, Weeks, & Sterne 2011). Stratified analyses will be conducted according to impulse control disorder and substance use disorders, length of intervention (duration time) and doses.

To address the variability in drug dose, and potential differences between comparator groups which all add to the complexity of the evidence base the mixed treatment comparisons, will be used to estimate the risk of adverse events, while acknowledging and modelling the complexity of the structure of the evidence base (Warren et al. 2014) .

We will use the non-central hypergeometric distribution likelihood leading to mixed-effects conditional logistic regression - an approximate inference technique for generalized linear mixed models (Platt et al. 1999). For incidence rate ratio meta-analysis, it leads to random effects logistic regression with an offset variable (Stijnen et al. 2010). The generalised linear mixed model (GLMM) will be performed using SAS procedures for fitting models to non-normal or normal data with correlations or nonconstant variability (Stijnen, Hamza, & Ozdemir 2010).

Our main analysis will be based on a network meta-analysis model applied to the dataset, in which each drug (i.e. nalmefene, placebo and comparator) is treated distinctly, but different dose levels are ignored (Warren, Abrams, & Sutton 2014). Second, we will distinguish distinct nalmefene nodes by its dose; all nalmefene interventions will be compared against a placebo and a non-nalmefene control, as in previous models (Warren, Abrams, & Sutton 2014).

## Risk of bias across studies

The risk of bias across RCTs will be assessed by using stratified analyses for each of the domains included in the risk-of-bias tool, as recommended by the Cochrane Collaboration (Higgins, Altman, Gotzsche, Juni, Moher, Oxman, Savovic, Schulz, Weeks, & Sterne 2011).

# ETHICS AND DISSEMINATION

As no primary data collection will be undertaken, no additional formal ethical assessment and informed consent are required. Using data from randomised trials, this study will evaluate different nalmefene strategies for substance use disorders and impulse control disorders. Our goal is to help decision-makers and nalmefene drug prescribing physicians make evidence-based decisions, which will enable a comprehensive interpretation of the data for harm. Our review will present data for all nalmefene drug treatments, provide relative estimates of tolerability and potential harm, and evaluate the quality of the evidence in a thorough and consistent manner using the GRADE approach (Guyatt et al. 2011). The review will help facilitate evidence-based management. Miss Karina G. V. Johansen will draft the paper describing the results of the systematic review and meta-analysis, which will be disseminated by peer-review publication and conference presentation.

## Contributors

KGVJ, ST, AA, HL, AKP and RC participated in the conception and design of this protocol, including search strategy development. KGVJ and ST participated in search strategy development and will perform the search and selection in collaboration with RC. KGVJ and ST will retrieve the data. ST and RC provided statistical advice for the design. All authors will draft and critically review the manuscript and approve the final version.

## Funding

The development of this protocol was funded in part by unrestricted grants from the Oak Foundation (supporting The Parker Institute). The sponsor of the study will have no role in study design, data collection, data analysis, data interpretation, or writing of the report. The corresponding author will have full access to all the data in the study and have the final responsibility for the decision to submit for publication.

## Competing interests

KGVJ: None.

ST: None.

AA: None.

HL: None.

AKP: None

RC: None. He declares that he is involved in many health-care initiatives and research that could benefit from wide uptake of this publication (including Cochrane, and the GRADE Working Group).

*Musculoskeletal Statistics Unit, The Parker Institute is grateful for the financial support received from public and private foundations, companies and private individuals over the years.*

*The Parker Institute is supported by a core grant from the Oak Foundation; The Oak Foundation is a group of philanthropic organizations that, since its establishment in 1983, has given grants to not-for-profit organizations around the world.*

References

American Psychiatric Association 2013. *Diagnostic and statistical manual of mental disorders: DSM-5*, 5. ed ed.

Christensen, R., Kristensen, P.K., Bartels, E.M., Bliddal, H., & Astrup, A. 2007. Efficacy and safety of the weight-loss drug rimonabant: a meta-analysis of randomised trials. *Lancet*, 370, (9600) 1706-1713 available from: PM:18022033

Cochran, G.W. 1954. The combination of estimates from different experiments. *Biometrics*, 10, 101-129

Cottone, P., Sabino, V., Steardo, L., & Zorrilla, E.P. 2008. Opioid-dependent anticipatory negative contrast and binge-like eating in rats with limited access to highly preferred food. *Neuropsychopharmacology*, 33, (3) 524-535 available from: PM:17443124

de, Z.M. & Mitchell, J.E. 1992. Opiate antagonists and eating behavior in humans: a review. *J.Clin.Pharmacol.*, 32, (12) 1060-1072 available from: PM:1487543

Drobes, D.J., Anton, R.F., Thomas, S.E., & Voronin, K. 2003. A clinical laboratory paradigm for evaluating medication effects on alcohol consumption: naltrexone and nalmefene. *Neuropsychopharmacology*, 28, (4) 755-764 available from: PM:12655322

European Medicines Agency 2012, *Assessement report Selincro*, European Medicines Agency, United Kingdom.

European Medicines Agency 2014, *summery of product characteristics*.

Fudala, P.J., Heishman, S.J., Henningfield, J.E., & Johnson, R.E. 1991. Human pharmacology and abuse potential of nalmefene. *Clin.Pharmacol.Ther.*, 49, (3) 300-306 available from: PM:2007323

Grant, J.E., Odlaug, B.L., Potenza, M.N., Hollander, E., & Kim, S.W. 2010. Nalmefene in the treatment of pathological gambling: multicentre, double-blind, placebo-controlled study. *Br.J.Psychiatry*, 197, (4) 330-331 available from: PM:20884959

Grant, J.E. & Potenza, M.N. 2004. Impulse control disorders: clinical characteristics and pharmacological management. *Ann.Clin.Psychiatry*, 16, (1) 27-34 available from: PM:15147110

Grant, J.E., Potenza, M.N., Hollander, E., Cunningham-Williams, R., Nurminen, T., Smits, G., & Kallio, A. 2006. Multicenter investigation of the opioid antagonist nalmefene in the treatment of pathological gambling. *Am.J.Psychiatry*, 163, (2) 303-312 available from: PM:16449486

Gual, A., He, Y., Torup, L., van den Brink, W., & Mann, K. 2013. A randomised, double-blind, placebo-controlled, efficacy study of nalmefene, as-needed use, in patients with alcohol dependence. *Eur.Neuropsychopharmacol.*, 23, (11) 1432-1442 available from: PM:23562264

Guyatt, G.H., Oxman, A.D., Schunemann, H.J., Tugwell, P., & Knottnerus, A. 2011. GRADE guidelines: a new series of articles in the Journal of Clinical Epidemiology. *J.Clin.Epidemiol.*, 64, (4) 380-382 available from: PM:21185693

Higgins, J.P., Altman, D.G., Gotzsche, P.C., Juni, P., Moher, D., Oxman, A.D., Savovic, J., Schulz, K.F., Weeks, L., & Sterne, J.A. 2011. The Cochrane Collaboration's tool for assessing risk of bias in randomised trials. *BMJ*, 343, d5928 available from: PM:22008217

Ioannidis, J.P., Evans, S.J., Gotzsche, P.C., O'Neill, R.T., Altman, D.G., Schulz, K., & Moher, D. 2004. Better reporting of harms in randomized trials: an extension of the CONSORT statement. *Ann.Intern.Med.*, 141, (10) 781-788 available from: PM:15545678

Karhuvaara, S., Simojoki, K., Virta, A., Rosberg, M., Loyttyniemi, E., Nurminen, T., Kallio, A., & Makela, R. 2007. Targeted nalmefene with simple medical management in the treatment of heavy drinkers: a randomized double-blind placebo-controlled multicenter study. *Alcohol Clin.Exp.Res.*, 31, (7) 1179-1187 available from: PM:17451401

Kelly, E., Mundell, S.J., Sava, A., Roth, A.L., Felici, A., Maltby, K., Nathan, P.J., Bullmore, E.T., & Henderson, G. 2014. The opioid receptor pharmacology of GSK1521498 compared to other ligands with differential effects on compulsive reward-related behaviours. *Psychopharmacology (Berl)* available from: PM:24973897

Lazarou, J., Pomeranz, B.H., & Corey, P.N. 1998. Incidence of adverse drug reactions in hospitalized patients: a meta-analysis of prospective studies. *JAMA*, 279, (15) 1200-1205 available from: PM:9555760

Liberati, A., Altman, D.G., Tetzlaff, J., Mulrow, C., Gotzsche, P.C., Ioannidis, J.P., Clarke, M., Devereaux, P.J., Kleijnen, J., & Moher, D. 2009. The PRISMA statement for reporting systematic reviews and meta-analyses of studies that evaluate health care interventions: explanation and elaboration. *J.Clin.Epidemiol.*, 62, (10) e1-34 available from: PM:19631507

Mason, B.J., Ritvo, E.C., Morgan, R.O., Salvato, F.R., Goldberg, G., Welch, B., & Mantero-Atienza, E. 1994. A double-blind, placebo-controlled pilot study to evaluate the efficacy and safety of oral nalmefene HCl for alcohol dependence. *Alcohol Clin.Exp.Res.*, 18, (5) 1162-1167 available from: PM:7847600

Nissen, S.E. & Wolski, K. 2007. Effect of rosiglitazone on the risk of myocardial infarction and death from cardiovascular causes. *N.Engl.J.Med.*, 356, (24) 2457-2471 available from: PM:17517853

Pirmohamed, M., James, S., Meakin, S., Green, C., Scott, A.K., Walley, T.J., Farrar, K., Park, B.K., & Breckenridge, A.M. 2004. Adverse drug reactions as cause of admission to hospital: prospective analysis of 18 820 patients. *BMJ*, 329, (7456) 15-19 available from: PM:15231615

Platt, R.W., Leroux, B.G., & Breslow, N. 1999. Generalized linear mixed models for meta-analysis. *Stat.Med.*, 18, (6) 643-654 available from: PM:10204195

Sadock, J.B. & Sadock, A.V. 2008. *Kaplan and Sadock´s concise textbook of clinical psychiatry*, 3 ed. philadelphia, Wolters Kluwer Lippincott Williams & Wilkins.

Schreiber, L., Odlaug, B.L., & Grant, J.E. 2011. Impulse control disorders: updated review of clinical characteristics and pharmacological management. *Front Psychiatry*, 2, 1 available from: PM:21556272

Soyka, M. & Rosner, S. 2010. Nalmefene for treatment of alcohol dependence. *Expert.Opin.Investig.Drugs*, 19, (11) 1451-1459 available from: PM:20868291

Stijnen, T., Hamza, T.H., & Ozdemir, P. 2010. Random effects meta-analysis of event outcome in the framework of the generalized linear mixed model with applications in sparse data. *Stat.Med.*, 29, (29) 3046-3067 available from: PM:20827667

van der Hooft, C.S., Dieleman, J.P., Siemes, C., Aarnoudse, A.J., Verhamme, K.M., Stricker, B.H., & Sturkenboom, M.C. 2008. Adverse drug reaction-related hospitalisations: a population-based cohort study. *Pharmacoepidemiol.Drug Saf*, 17, (4) 365-371 available from: PM:18302300

Warren, F.C., Abrams, K.R., & Sutton, A.J. 2014. Hierarchical network meta-analysis models to address sparsity of events and differing treatment classifications with regard to adverse outcomes. *Stat.Med.*, 33, (14) 2449-2466 available from: PM:24623455

World Health Organization. ICD-10 Version:2014. 25-11-2014.
Ref Type: Online Source

# Appendix

## Table 1

| Reference | Year | Condition | Abstract | Result of serious adverse/ adverse event associated with use of nalmefene |
|---|---|---|---|---|
| Jonas DE, Amick HR, Feltner C, Bobashev G, Thomas K, Wines R, Kim MM, Shanahan E, Gass CE, Rowe CJ, Garbutt JC.<br><br>Pharmacotherapy for Adults With Alcohol-Use Disorders in Outpatient Settings [Internet] | 2014 | Alcohol use-disorder | Emphasising effect of different drugs to reduce alcohol intake, drinking days and occasions.<br>135 studies were included.<br>7 studies compering nalmefene with placebo. | Mortality: two trials reported deaths. In one trial two in placebo group commit suicide.<br>In the other trial one patient allocated to the placebo group died of hepatocellular carcinoma and one patient randomized to nalmefene experienced sudden death of unknown cause.<br><br>In studies comparing nalmefene with placebo, four cases of suicide attempts or suicidal ideation in nalmefene group and 9 in placebo group were reported.<br><br>**Adverse events:**<br>The meta-analyses found that patients treated with nalmefene had a higher risk of withdrawal due to adverse events, compared with patients who received placebo. |
| Jonas DE, Amick HR, Feltner C, Bobashev G, Thomas K, Wines R, Kim MM, Shanahan E, Gass CE, Rowe CJ, Garbutt JC.<br><br>Pharmacotherapy for adults with alcohol use disorders in outpatient settings: a systematic review and meta-analysis | 2014 | Alcohol use disorder | 122 RCT and 1 cohort study was included.<br>One study comparing nalmefene with placebo.The meta-analysis emphasising the effect of different drugs to reduce alcohol intake, drinking days and occasions.<br>A section with adverse effects was conducted. | Compared with placebo, patients treated with naltrexone or nalmefene had a higher risk of adverse events.<br><br>Patients allocated to the nalmefene group had a higher risk of dizziness, headache insomnia, nausea and vomiting compare to patients allocated to the placebo group. |

| | | | | |
|---|---|---|---|---|
| | | | | |
| Gowing L, Ali R, White JM.<br><br>Opioid antagonists under heavy sedation or anaesthesia for opioid withdrawal. | 2010 | Withdrawal treatment | Nine studies met the inclusion criteria eight were RCT.<br>Different drugs was investigated, including one study investigating nalmefene, as part of a anaesthesia group | In the study including nalmefene as part of anaesthesia group three potentially life-threatening adverse events, all in the anaesthesia group were reported.<br>There is a significantly increased risk of serious adverse events with anaesthesia-assisted approaches. |
| Srisurapanont M, Jarusuraisin N.<br><br>Opioid antagonist for alcohol dependence | 2005 | Alcohol dependence. | 29 RCT were included, two studies included nalmefene.<br><br>Effectiveness of opioid antagonists in compression of placebo, other medications and psychosocial treatment. | Adverse or serious adverse event wasn't included regarding nalmefene. |
| Rösner S, Hackl-Herrwerth A, Leucht S, Vecchi S, Srisurapanont M, Soyka M.<br><br>Opioid antagonists for alcohol dependence. | 2010 | Alcohol dependence | 29 RCT were included, two studies included nalmefene compared to placebo.<br><br>Effectiveness of different opioid antagonists. | Adverse or serious adverse event wasn't included regarding nalmefene |

| | | | | |
|---|---|---|---|---|
| Mann k.<br><br>Pharmacotherapy of alcohol dependence: a review of the clinical data. | 2004 | Alcohol dependence | A review update of different drugs for alcohol dependence:<br>Current therapies:<br>Disulfram<br>Naltrexone<br>Acamprosate<br><br>Experimental drugs:<br>Nalmefene<br>Dopaminergic drugs<br>Serotonergic drugs<br>TCA<br>Antimanic drugs<br>Sedative/anxiolytics | In the section of safety, only current therapies were mentioned. |
| Kjellberg F, Tramèr MR.<br><br>Pharmacological control of opioid-induced pruritus: a quantitative systematic review of randomized trials. | 2001 | Opioid-induced pruritus | 14 studies were included.<br>1 trial included nalmefene iv.<br><br>Effectiveness of different drugs on pruritus. | Intravenous nalmefene 0.5 or 1 mg was not anti-pruritic.<br><br>Adverse or serious adverse events wasn't reported |
| Keating GM. Nalmefene: a review of its use in the treatment of alcohol dependence | 2013 | Alcohol dependence | Two studies were included; ESENSE 1 and ESENSE 2<br>The study investigated efficacy and tolerability of as-needed oral nalmefene in the treatment of alcohol dependence | Adverse events occurred 81,5 % in nalmefene group and 66,9% in the placebo group in the ESENSE 1. Adverse events occurred 68,0 % in nalmefene group and 59,1% in the placebo group in the ESENSE 2.<br><br>Adverse events included dizziness, nausea, fatigue, headache, nasopharyngitis, sleep disorder, insomnia, vomiting, hyperhidrosism, decreased appetite, somnolence and tachycardia.<br><br>Adverse events tended to emerge shortly within 1 day after administration of the first dose of study medication. |

| | | | | |
|---|---|---|---|---|
| | | | | Among nalmefene and placebo patients, serious adverse events were reported.<br>In the nalmefene group 5.9% and in the placebo group 6.7 % of patients, in the ESENSE 1 trial reported serious adverse events.<br>In the nalmefene group 2.2% and in the placebo group 4.7% of patients, in the ESENSE 2 trial reported serious adverse events.<br>In the nalmefene group 6.9% and in the placebo group 5.4% of patients, in the SENSE trial reported serious adverse events. |
