# Supplementary material for: Harms associated with taking nalmefene for substance use and impulse control disorders: A systematic review and meta-analysis of randomised controlled trials
Source: PLoS One. 2017 Aug 29;12(8):e0183821. doi: 10.1371/journal.pone.0183821 (PMC5574613; doi:10.1371/journal.pone.0183821)
Supplement: S2 File — (DOCX) [file pone.0183821.s002.docx]

**S2 File. Reference list of all 31 full text papers included based on title and abstract.**

11 full-text articles excluded:

*Not English publication: 1*

Maremmani I, Presta S, Petracca A, Di NM, Maremmani AGI, Ruggeri F, et al. Nalmefene: Clinical and real world evidence in the treatment of alcohol dependence. Journal of Psychopathology 201420(1):80-91.

*Review*: 1

Paille F, Martini H. Nalmefene: a new approach to the treatment of alcohol dependence. Subst Abuse Rehabil 2014;5:87-94.

*Not Randomised controlled trial: 6*

De FS, Porrari R, Zingaretti P, Vitale M, Di LA, De PS. Nalmefene, comparison between two groups: In steady and as-needed assumption. European neuropsychopharmacology 201424:S669.

Laramee P, Brodtkorb TH, Rahhali N, Knight C, Barbosa C, Francois C, et al. The cost-effectiveness and public health benefit of nalmefene added to psychosocial support for the reduction of alcohol consumption in alcohol-dependent patients with high/very high drinking risk levels: a Markov model. BMJ Open 2014;4(9):e005376.

Martinotti G, Di GM, Di NM, Janiri L. Nalmefene in dual diagnosed alcoholics: An open study. European neuropsychopharmacology 201424:S669-S670.

Nash CB, Caldwell RV, Tuttle RR.Studies on nalmefene, an opioid antagonist. FED PROC 198443(4):no-3987.

Salvato FR, Mason BJ. Changes in transaminases over the course of a 12-week, double-blindnalmefene trial in a 38-year-old female subject. Alcohol ClinExp Res 1994 Oct;18(5):1187-9.

Vasile D, Vasiliu O, Sopterean GA, Bratu RE, Androne F, Vasile F. Effectiveness and tolerability of nalmefene in alcohol use dependence comorbid with schizophrenia. European neuropsychopharmacology 201424:S538.

*Not nalmefene Randomised controlled trial:1*

*Mills IH, Park GR, Manara AR, Merriman RJ. Treatment of compulsive behaviour in eating disorders with intermittent ketamine infusions. QJM 1998 Jul;91(7):493-503.*

*Other: 1*

Nature Reviews Drug Discovery. Trial watch: Nalmefene reduces alcohol use in phase III trial. Nat Rev Drug Discov 2011 Aug;10(8):566.

*Not substance use or impulse control disorder: 1*

Dixon R, Howes J, Garg D. Nalmefene: Pharmacokinetics and tolerance of a new orally active narcotic antagonist. FED PROC 198544(5):No-7670.

**5 trials excluded from the systematic review:**

*Pooled analysis of included randomised controlled trial: 2*

Grant JE, Kim SW, Hollander E, Potenza MN. Predicting response to opiate antagonists and placebo in the treatment of pathological gambling. Psychopharmacology (Berl) 2008 Nov;200(4):521-7.

van den Brink W, Aubin HJ, Bladstrom A, Torup L, Gual A, Mann K. Efficacy of as-needed nalmefene in alcohol-dependent patients with at least a high drinking risk level: results from a subgroup analysis of two randomized controlled 6-month studies. Alcohol Alcohol 2013 Sep;48(5):570-8.

*Spin of included randomised controlled trial: 2*

Arias AJ, Armeli S, Gelernter J, Covault J, Kallio A, Karhuvaara S, et al. Effects of opioid receptor gene variation on targeted nalmefene treatment in heavy drinkers. Alcohol ClinExp Res 2008 Jul;32(7):1159-66.

Drobes DJ, Anton RF, Thomas SE, Voronin K. Effects of naltrexone and nalmefene on subjective response to alcohol among non-treatment-seeking alcoholics and social drinkers. Alcohol ClinExp Res 2004 Sep;28(9):1362-70.

*Study design was not a randomised controlled trial: 1*

Jones HE, Johnson RE, Fudala PJ, Henningfield JE, Heishman SJ. Nalmefene: blockade of intravenous morphine challenge effects in opioid abusing humans. Drug Alcohol Depend 2000 Jul 1;60(1):29-37.

**15 randomised controlled trials included in the systematic review:**

Anton RF, Pettinati H, Zweben A, Kranzler HR, Johnson B, Bohn MJ, et al. A multi-site dose ranging study of nalmefene in the treatment of alcohol dependence. J ClinPsychopharmacol 2004 Aug;24(4):421-8.

Drobes DJ, Anton RF, Thomas SE, Voronin K. A clinical laboratory paradigm for evaluating medication effects on alcohol consumption: naltrexone and nalmefene. Neuropsychopharmacology 2003 Apr;28(4):755-64.

Drobes DJ, Anton RF. Drinking in alcoholics following an alcohol challenge research protocol. J Stud Alcohol 2000 Mar;61(2):220-4.

Edison Investment Research.Biotie Therapies Corp. Update 21 June 2011. 2011.

Edison Investment Research.Biotie Therapies Corp. Update 21 June 2011. 2011.

Grant JE, Odlaug BL, Potenza MN, Hollander E, Kim SW. Nalmefene in the treatment of pathological gambling: multicentre, double-blind, placebo-controlled study. Br J Psychiatry 2010 Oct;197(4):330-1.

Grant JE, Potenza MN, Hollander E, Cunningham-Williams R, Nurminen T, Smits G, et al. Multicenter investigation of the opioid antagonist nalmefene in the treatment of pathological gambling. Am J Psychiatry 2006 Feb;163(2):303-12.

Gual A, He Y, Torup L, van den Brink W, Mann K. A randomised, double-blind, placebo-controlled, efficacy study of nalmefene, as-needed use, in patients with alcohol dependence. EurNeuropsychopharmacol 2013 Nov;23(11):1432-42.

H.Lundbeck A/S. Effects of Nalmefene After Single Dose on the Blood Oxygen Level Dependent (BOLD) fMRI Signal in the Ventral Striatum to Reward Responding in the Monetary Incentive Delay Task (MIDT), in Non-treatment Seeking Subjects With Alcohol Dependence Following Alcohol Challenge. 2015.

Karhuvaara S, Simojoki K, Virta A, Rosberg M, Loyttyniemi E, Nurminen T, et al. Targeted nalmefene with simple medical management in the treatment of heavy drinkers: a randomized double-blind placebo-controlled multicenter study. Alcohol ClinExp Res 2007 Jul;31(7):1179-87.

Mann K, Bladstrom A, Torup L, Gual A, van den Brink W. Extending the treatment options in alcohol dependence: a randomized controlled study of as-needed nalmefene. Biol Psychiatry 2013 Apr 15;73(8):706-13.

Mason BJ, Ritvo EC, Morgan RO, Salvato FR, Goldberg G, Welch B, et al. A double-blind, placebo-controlled pilot study to evaluate the efficacy and safety of oral nalmefeneHCl for alcohol dependence. Alcohol ClinExp Res 1994 Oct;18(5):1162-7.

Mason BJ, Salvato FR, Williams LD, Ritvo EC, Cutler RB. A double-blind, placebo-controlled study of oral nalmefene for alcohol dependence. Arch Gen Psychiatry 1999 Aug;56(8):719-24.

Somaxon Pharmaceuticals I. Somaxon Pharmaceuticals Reports Positive Results From a Pilot Phase 2 Study of Oral Nalmefene in Smoking Cessation. 31-1-2015.

van den Brink W, Sorensen P, Torup L, Mann K, Gual A. Long-term efficacy, tolerability and safety of nalmefene as-needed in patients with alcohol dependence: A 1-year, randomised controlled study. J Psychopharmacol 2014 Mar 26;28(8):733-44.
