# Supplementary material for: Harms associated with taking nalmefene for substance use and impulse control disorders: A systematic review and meta-analysis of randomised controlled trials
Source: PLoS One. 2017 Aug 29;12(8):e0183821. doi: 10.1371/journal.pone.0183821 (PMC5574613; doi:10.1371/journal.pone.0183821)
Supplement: S1 Table — (DOCX) [file pone.0183821.s004.docx]

**S1 Table exploratory table of all serious adverse events reported in the included studies**

| **Authors, year**  **Disorders** | **Gual et al. 2013** | | **Mann et al. 2013** | | **Van Den Brink et al.**  **2014** | | **Karhuvaara et al.**  **2007** | |
| --- | --- | --- | --- | --- | --- | --- | --- | --- |
| **Serious adverse events** | Placebo | Nalmefene | Placebo | Nalmefene | Placebo | Nalmefene | Placebo | Nalmefene |
| **Cardiac disorders** |  |  |  |  |  |  |  |  |
| *Cardiac arrest* |  |  | 1 |  |  |  |  |  |
| *Myocardial infarction* |  | 1 |  |  |  |  |  |  |
| *Ventricular extrasystoles* |  |  |  |  |  | 1 |  |  |
| *Atrial fibrillation* |  |  |  |  |  | 2 |  |  |
| **Congenital, familial, and genetic disorders** |  |  |  |  |  |  |  |  |
| *Adenomatous polyposis coli* |  |  |  |  |  | 1 |  |  |
| **Eye disorders** |  |  |  |  |  |  |  |  |
| *Diplopia* |  |  |  |  |  | 1 |  |  |
| **Gastrointestinal disorders** |  |  |  |  |  |  |  |  |
| *Hemorrhoids* |  |  | 1 |  |  |  |  |  |
| *Gastric ulcer perforation* |  |  |  | 1 |  |  |  |  |
| *Pancreatitis acute* | 1 |  |  |  |  |  |  |  |
| *Alcohol pancreatitis* |  |  |  |  |  |  |  | 1 |
| *Rectal hemorrhage* | 1 |  |  |  |  |  |  |  |
| *Crohn's disease* |  |  |  |  | 1 |  |  |  |
| *Diverticulum intestinal* |  |  |  |  | 1 |  |  |  |
| *Large intestine perforation* |  |  |  |  |  | 1 |  |  |
| *Esophagitis ulcerative* |  |  |  |  |  | 1 |  |  |
| **General disorders** |  |  |  |  |  |  |  |  |
| *Non-cardiac chest pain* |  |  |  | 1 |  |  |  |  |
| *Sudden death* |  | 1 |  |  |  |  |  |  |
| **Hepatobiliary disorders** |  |  |  |  |  |  |  |  |
| *Liver disorder* |  |  |  |  | 1 |  |  |  |
| *Biliary colic* |  |  | 1 |  |  |  |  |  |
| *Cholecystitis acute* |  |  | 1 |  |  |  |  |  |
| **Infections and infestations** |  |  |  |  |  |  |  |  |
| *Bronchopneumonia* |  |  |  |  |  | 1 |  |  |
| *Abscess limb* |  |  |  |  | 1 |  |  |  |
| *Pneumonia* | 1 |  |  |  | 1 | 1 |  |  |
| *Pulmonary tuberculosis* |  |  |  |  |  | 1 |  |  |
| *Pyelonephritis* |  |  |  |  | 1 |  |  |  |
| *Pyothorax* | 1 |  |  |  | 1 |  |  |  |
| *Subcutaneous abscess* | 1 |  |  |  |  |  |  |  |
| *Postoperative wound infection* |  |  | 1 |  |  |  |  |  |
| **Injury, poisoning, and procedural complications** |  |  |  |  |  |  |  |  |
| *Accidental overdose* | 1 |  |  |  |  |  |  |  |
| *Alcohol poisoning* | 1 |  | 1 | 1 |  | 1 |  |  |
| *Drug toxicity* | 1 |  |  |  |  |  |  |  |
| *Ankle fracture* |  |  |  | 1 |  |  |  |  |
| *Fall* |  | 1 | 1 |  |  | 2 |  |  |
| *Femoral neck fracture* |  |  |  | 1 |  |  |  |  |
| *Fibula fracture* | 1 |  |  | 1 | 1 |  |  |  |
| *Ligament rupture* |  |  |  |  |  | 1 |  |  |
| *Hand fracture* |  |  |  | 1 |  |  |  |  |
| *Humerus fracture* |  |  | 1 |  |  |  |  |  |
| *Multiple fracture* |  |  | 1 |  |  |  |  |  |
| *Rib fracture* |  |  | 1 |  | 1 |  |  |  |
| *Tibia fracture* |  |  |  |  | 1 |  |  |  |
| *Traumatic brain injury* |  |  |  |  |  | 1 |  |  |
| *Ulna fracture* |  |  |  |  |  | 1 |  |  |
| *Upper limb fracture* |  |  |  |  |  | 1 |  |  |
| *Head injury* |  | 1 |  |  |  |  |  |  |
| *Intentional overdose* | 3 |  |  |  |  |  |  |  |
| *Tendon rupture* |  | 1 |  |  |  |  |  |  |
| *Road traffic accident* |  |  | 1 |  |  |  |  |  |
| *Subdural hematoma* |  |  | 1 |  |  |  |  |  |
| **Investigations** |  |  |  |  |  |  |  |  |
| *Blood lactic acid increased* |  |  | 1 |  |  |  |  |  |
| *Blood potassium increased* |  |  | 1 |  |  |  |  |  |
| *Blood sodium decreased* |  |  | 1 |  |  |  |  |  |
| *Tibia fracture* | 1 |  |  |  |  |  |  |  |
| **Musculoskeletal and connective tissue disorders** |  |  |  |  |  |  |  |  |
| *Back pain* |  |  |  | 1 |  |  |  |  |
| *Intervertebral disc protrusion* |  |  |  | 1 |  |  |  |  |
| *Osteoarthritis* |  |  | 1 |  |  |  |  |  |
| *Pain in extremity* |  |  |  | 1 |  |  |  |  |
| *Polyarthritis* |  |  |  | 1 |  |  |  |  |
| *Tendonitis* |  |  |  | 1 |  |  |  |  |
| **Metabolism and nutrition disorders** |  |  |  |  |  |  |  |  |
| *Decreased appetite* |  |  |  |  |  | 1 |  |  |
| **Neoplasms benign, malignant, and unspecified (incl. cysts and polyps)** |  |  |  |  |  |  |  |  |
| *Laryngeal cancer* |  |  | 1 |  |  |  |  |  |
| *Malignant melanoma* |  |  | 1 |  |  |  |  |  |
| *Esophageal carcinoma* |  |  |  |  |  | 1 |  |  |
| *Prostate cancer* |  |  |  |  |  | 1 |  |  |
| *Bile duct cancer* | 1 |  |  |  |  |  |  |  |
| **Nervous system disorders** |  |  |  |  |  |  |  |  |
| *Convulsion* |  |  | 2 |  |  |  |  |  |
| *Dizziness* | 1 |  |  | 1 |  |  |  |  |
| *Epilepsy* | 1 |  |  |  |  | 1 |  |  |
| *Balance disorder* |  |  |  |  |  | 1 |  |  |
| *Headache* |  |  |  |  |  | 1 |  |  |
| *Syncope* |  |  |  |  |  | 1 |  |  |
| *Subarachnoid hemorrhage* |  | 1 |  |  |  |  |  |  |
| **Psychiatric disorders** |  |  |  |  |  |  |  |  |
| *Alcohol abuse* |  |  | 1 |  | 1 | 1 |  |  |
| *Alcohol withdrawal syndrome* |  |  |  |  | 1 | 9 |  |  |
| *Alcoholism* |  | 2 | 2 | 2 |  | 1 |  |  |
| *Alcoholic hangover* |  |  |  |  |  | 1 |  |  |
| *Anorexia nervosa* |  |  |  |  |  | 1 |  |  |
| *Anxiety* |  |  |  |  |  | 1 |  |  |
| *Completed suicide* |  |  | 2 |  |  |  |  |  |
| *Depression* |  |  |  | 1 |  | 1 |  |  |
| *Disorientation* |  |  |  |  |  | 2 |  |  |
| *Insomnia* |  |  |  |  |  | 1 |  |  |
| *Nightmare* |  |  |  |  |  | 1 |  |  |
| *Suicidal behaviour* | 1 |  |  |  |  | 1 |  |  |
| **Renal and urinary disorders** |  |  |  |  |  |  |  |  |
| *Renal failure* |  |  | 1 |  |  |  |  |  |
| **Respiratory, thoracic, and mediastinal disorders** |  |  |  |  |  |  |  |  |
| *Epiglottic cyst* |  |  |  | 1 |  |  |  |  |
| *Epistaxis* |  |  |  | 1 |  |  |  |  |
| *Pneumothorax* |  |  | 1 |  | 1 |  |  |  |
| **Vascular disorders** |  |  |  |  |  |  |  |  |
| *Arteritis* | 1 |  |  |  |  | 1 |  |  |
| *Hypotension* | 1 |  | 1 |  | 1 |  |  |  |
