# Supplementary material for: Harms associated with taking nalmefene for substance use and impulse control disorders: A systematic review and meta-analysis of randomised controlled trials
Source: PLoS One. 2017 Aug 29;12(8):e0183821. doi: 10.1371/journal.pone.0183821 (PMC5574613; doi:10.1371/journal.pone.0183821)
Supplement: S2 Table — * NE = not estimable. NA = Not applicable. (DOCX) [file pone.0183821.s005.docx]

| **Variable** | **Trials**  **(No.)** | **Peto´s OR** | **95% CI** | **I^2^** | ***P* for heterogeneity** |
| --- | --- | --- | --- | --- | --- |
| All trials | 8 | 0.97 | 0.64, 1.44 | 0.18 | 0.30 |
| **Sequence generation** |  |  |  | NA | NA |
| Adequate | 5 | 0.97 | 0.64, 1.44 | 0.18 | 0.30 |
| Unclear | 3 | NE | NE | NA | NA |
| Inadequate | 0 | NE | NE | NA | NA |
| **Allocation concealment** |  |  |  | NA | NA |
| Adequate | 6 | 0.97 | 0.64, 1.44 | 0.18 | 0.30 |
| Unclear | 2 | NE | NE | NA | NA |
| Inadequate | 0 | NE | NE | NA | NA |
| **Blinding** |  |  |  | NA | NA |
| Adequate | 4 | 0.97 | 0.64, 1.44 | 0.18 | 0.30 |
| Unclear | 4 | NE | NE | NA | NA |
| Inadequate | 0 | NE | NE | NA | NA |
| **ITT** |  |  |  | 0 | 0.57 |
| Adequate | 5 | 1.33 | 0.41, 4.28 | NA | NA |
| Unclear | 0 | NE | NE | NA | NA |
| Inadequate | 3 | 0.92 | 0.60, 1.42 | 0.40 | 0.19 |
| **Incomplete outcome** |  |  |  | 0 | 0.57 |
| Adequate | 2 | 1.33 | 0.41, 4.28 | NA | NA |
| Unclear | 3 | NE | NE | NA | NA |
| Inadequate | 3 | 0.92 | 0.60, 1.42 | 0.40 | 0.19 |
| **Selective outcome** |  |  |  | 0 | 0.57 |
| Adequate | 5 | 0.92 | 0.60, 1.42 | 0.40 | 0.19 |
| Unclear | 1 | 1.33 | 0.41, 4.28 | NA | NA |
| Inadequate | 2 | NE | NE | NA | NA |
