# Supplementary material for: Harms associated with taking nalmefene for substance use and impulse control disorders: A systematic review and meta-analysis of randomised controlled trials
Source: PLoS One. 2017 Aug 29;12(8):e0183821. doi: 10.1371/journal.pone.0183821 (PMC5574613; doi:10.1371/journal.pone.0183821)
Supplement: S3 Table — (DOCX) [file pone.0183821.s006.docx]

| **Study** |  | **Serious adverse event** | **Withdrawal due to adverse events** | **Psychiatric serious adverse events** | **Depression** | **Anxiety** | **All-cause mortality** | **total** |
| --- | --- | --- | --- | --- | --- | --- | --- | --- |
| **Anton RF et al. 2004** | **Nalmefene** | NA | 21 | NA | NA | NA | NA | 202 |
|  | **Placebo** | NA | 2 | NA | NA | NA | NA | 68 |
| **Grant JE et al. 2006** | **Nalmefene** | NA | 50 | NA | NA | NA | NA | 156 |
|  | **Placebo** | NA | 3 | NA | NA | NA | NA | 51 |
| **Gual A et al. 2013** | **Nalmefene** | 7 | 15 | 1 | 0 | 0 | 1 | 341 |
|  | **Placebo** | 14 | 8 | 2 | 0 | 0 | 0 | 337 |
| **Karhuvaara S et al. 2007** | **Nalmefene** | 8 | 38 | NA | NA | NA | NA | 242 |
|  | **Placebo** | 4 | 3 | NA | NA | NA | NA | 161 |
| **Mann K et al. 2013** | **Nalmefene** | 17 | 62 | 3 | 1 | 0 | 0 | 302 |
|  | **Placebo** | 18 | 20 | 5 | 0 | 0 | 2 | 296 |
| **Mason BJ et al. 1994** | **Nalmefene** | 0 | 3 | NA | NA | NA | NA | 14 |
|  | **Placebo** | 0 | 1 | NA | NA | NA | NA | 7 |
| **Mason BJ et al. 1999** | **Nalmefene** | 0 | 3 | NA | NA | NA | NA | 70 |
|  | **Placebo** | 0 | 0 | NA | NA | NA | NA | 35 |
| **Van den brink et al 2014** | **Nalmefene** | 35 | 43 | 20 | 1 | 1 | 0 | 501 |
|  | **Placebo** | 8 | 2 | 2 | 0 | 0 | 0 | 164 |
