# Supplementary figures and images for: Harms associated with taking nalmefene for substance use and impulse control disorders: A systematic review and meta-analysis of randomised controlled trials
Source: PLoS One. 2017 Aug 29;12(8):e0183821. doi: 10.1371/journal.pone.0183821 (PMC5574613; doi:10.1371/journal.pone.0183821)

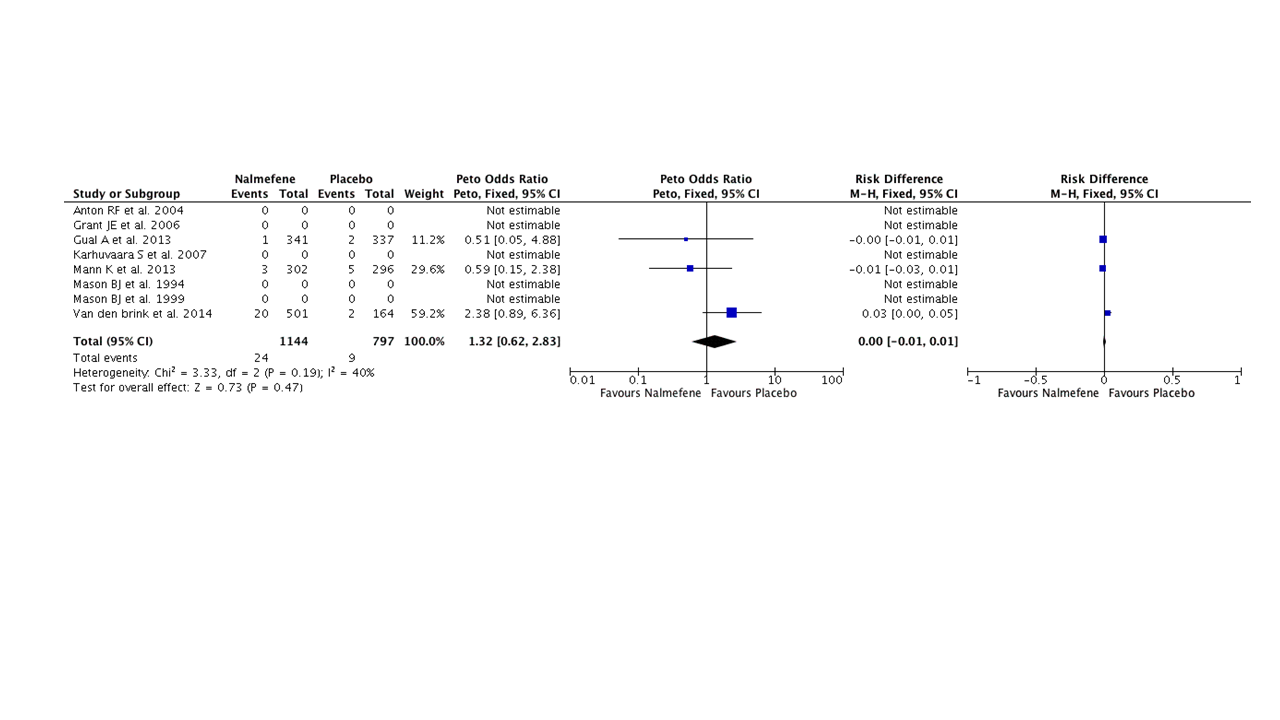

Supplement: S1 Fig — (TIF) [file pone.0183821.s007.tif]

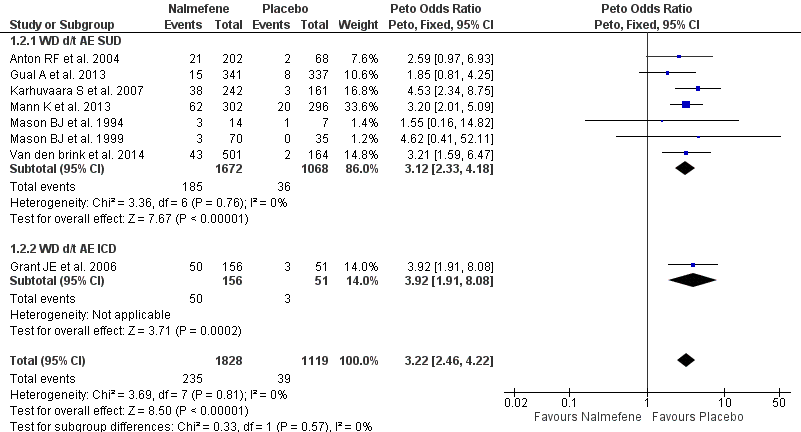

Supplement: S2 Fig — (PNG) [file pone.0183821.s008.png]
